# Aging and IGF-I: relationships with vitamin D and body composition. A mediation analysis

**DOI:** 10.3389/fnut.2025.1585696

**Published:** 2025-06-24

**Authors:** Roberto Vicinanza, Alessandro Frizza, Julie A. Pollard, Valentina Mazza, Massimo Ulderico De Martino, Giovanni Imbimbo, Pasquale Pignatelli, Evaristo Ettorre, Maria Del Ben, Alessio Molfino

**Affiliations:** ^1^Leonard Davis School of Gerontology, University of Southern California, Los Angeles, CA, United States; ^2^Department of Clinical, Internal, Anesthesiologic and Cardiovascular Sciences, Sapienza University of Rome, Rome, Italy; ^3^Department of Human, Philosophical and Educational Sciences, University of Salerno, Salerno, Italy; ^4^Department of Translational and Precision Medicine, Sapienza University of Rome, Rome, Italy

**Keywords:** aging, body composition, fat-free mass, frailty, IGF-I, insulin-like growth factor-I, multimorbidity, vitamin D

## Abstract

**Background:**

Insulin-like Growth Factor I (IGF-I) and Vitamin D are crucial for growth and metabolism, with their levels declining with age. However, their mutual interactions and contributions to body composition remain unclear.

**Objectives:**

To examine the relationships between IGF-I, Vitamin D, and body composition in geriatric outpatients, and to test the mediational role of IGF-I in the association between Vitamin D and Fat-Free Mass (FFM).

**Methods:**

One hundred thirty patients were eligible at the Geriatric Outpatient Clinic at the Policlinico Umberto I, Sapienza University of Rome, Italy. Multimorbidity was evaluated with the Cumulative Illness Rating scale for Geriatrics (CIRS-G). Body composition was measured using bioelectrical impedance analysis. Complete blood count, metabolic panel, IGF-I, and 25(OH) Vitamin D were assessed.

**Results:**

Ninety-one patients were included in the analysis. Mean age was 74.4 ± 7.2 years; 50.5% female. Mean BMI was 28 kg/m^2^ ± 3.9. Mean CIRS-G total score was 14.14 ± 4.1, and Severity Index (SI) was 1.16 ± 0.32. Median IGF-I was 122.0 ng/mL (IQR, 69.8) with higher levels in males compared to females (*p* = 0.0096). Mean 25(OH) Vitamin D was 27.04 ng/mL ± 14.69 with no significant sex difference. Level of 25(OH) Vitamin D positively correlated with IGF-I (*ρ* = 0.317, *p* = 0.003), while no correlation was found between Vitamin D and body composition parameters. Patients with higher IGF-I exhibited higher Total Body Water (TBW) (*p* = 0.024), Intracellular Water (ICW) (*p* = 0.018), FFM (*p* = 0.022), and Muscle Mass (MM) (*p* = 0.017), Body Cell Mass (BCM) (*p* = 0.046). Linear regression analysis showed that IGF-I and male sex predicted FFM (*B* = 13.933, *p* < 0.001; *B* = 0.040; *p* = 0.034; respectively). The mediation analysis confirmed no significant direct effect of Vitamin D on FFM (direct effect, *B* = −0.058, *p* = 0.319, 95% CI: −0.175, 0.058); however, the effect was significant when mediated by IGF-I (indirect effect, *B* = 0.039, SE = 0.022, 95% CI: 0.005, 0.091).

**Conclusion:**

These findings provide further evidence of a positive correlation between IGF-I and lean body mass and suggest that IGF-I may mediate the physiological effect of Vitamin D on FFM, highlighting their potential roles in assessing pre-frailty and personalizing nutrition interventions.

## 1 Introduction

Aging is a complex phenomenon characterized by the dynamic interplay among multiple biological processes, including alterations in mitochondrial function, loss of proteostasis, and dysregulated nutrient-sensing (1, 2). Over time, these mechanisms contribute to phenotypic changes such as modifications in body composition, which may increase the risk for sarcopenia and frailty (3–6). Reduced physical activity and age-related changes to metabolic and hormonal responses may further promote the loss of muscle mass (7–11). Insulin-like Growth Factor I (IGF-I) is a key regulator of growth and development and plays an essential role in the anabolic pathways (12, 13). IGF-I is primarily produced in the liver, where it mediates the actions of Growth Hormone (GH) (14, 15). Furthermore, IGF-I exerts autocrine and paracrine effects in muscle cells, promoting cell differentiation, survival, and repair (16–18). Upon binding to its transmembrane receptors, which consist of two extracellular α-subunits and two transmembrane β-subunits with intrinsic tyrosine kinase activity, similar to other hormones (19), IGF-I modulates several intracellular signaling cascades (20, 21). The PI3K/Akt and MAPK/ERK pathways modulate protein synthesis (22), muscle hypertrophy (23), and satellite cell activation (24, 25), contributing to muscle homeostasis.

IGF-I levels fluctuate throughout the lifespan, beginning with low concentrations in infancy, peaking during adolescence, and tending to decline in adulthood (26, 27). However, the biological role of IGF-I in the aging process is complex, with its effects on health outcomes influenced by metabolic changes and the presence of chronic illnesses (28–30). For example, low IGF-I has been associated with low HDL cholesterol (31), and reduced insulin sensitivity (32), both of which are known risk factors for cardiovascular disease (CVD) and type 2 diabetes mellitus (T2DM) (33–35). Additionally, low serum IGF-I has been linked to sarcopenia in both animal models and human studies (36, 37). In contrast, higher IGF-I levels have been associated with an increased risk of certain forms of cancer (28, 38).

A large prospective study involving over 7,000 individuals, stratified by age, described a U-shaped relationship between IGF-I levels and the risk of cancer, CVD, and all-cause mortality, suggesting that both low and high circulating IGF-I levels may increase the risk of such conditions (39). IGF-I levels are also influenced by factors such as physical activity and nutrient intake, further contributing to variability in research findings (40–44). In this regard, previous studies suggest a correlation between Vitamin D and IGF-I (45–48), although randomized controlled trials have yielded inconsistent results (49–51). Furthermore, the mechanisms underlying these observations remain elusive. Indeed, hepatocytes do not consistently express the nuclear Vitamin D receptor (VDR), while non-parenchymal liver cells have been shown to express VDR (52), suggesting a potential role of Vitamin D in modulating IGF-I bioavailability (53). Moreover, evidence suggests that IGF-I may stimulate the enzyme 1-*α*-hydroxylase in the kidneys, which converts 25-hydroxyvitamin D (25 OH Vitamin D) into its active form, calcitriol (1,25-dihydroxyvitamin D) (54, 55). In addition to the described effect of IGF-I on body composition, Vitamin D deficiency has been associated with depressive symptoms (56), frailty (57), muscle weakness (58), and an increased risk for falls (59, 60). Given the age-associated decline of both IGF-I and Vitamin D (27, 61), this study aimed to (i) examine the relationships between IGF-I, Vitamin D, and parameters of body composition; (ii) explore the clinical variables associated with IGF-I levels; and (iii) test IGF-I as a mediator in the relationship between Vitamin D and FFM in geriatric outpatients.

## 2 Materials and methods

### 2.1 Study design and criteria

This cross-sectional study was conducted at the Geriatric Outpatient Clinic at *Policlinico Umberto I University Hospital, Sapienza University of Rome, Italy*. Participants with the following criteria were considered eligible: age ≥65 years, written informed consent to participate in the study, no contraindications to body composition analysis according to the device manufacturer instructions, and independence in both the activities of daily living (ADL) and the instrumental activities of daily living (IADL). Exclusion criteria were: a body mass index (BMI) > 40 kg/m^2^, a history of alcohol misuse (more than 7 standard drinks per week for women and more than 14 standard drinks per week for men), alterations in liver enzymes such as alanine aminotransferase (ALT) and aspartate transaminase (AST) beyond the normal reference range, an estimated Glomerular Filtration Rate (eGFR) < 50 mL/min/1.73 m^2^, reported weight fluctuation in the past 3 months (>10% of total body weight), poorly controlled T2DM or individuals receiving insulin therapy, and history of cancer treated with radiation or chemotherapy in the past 5 years. Patients with acute medical conditions were excluded. A total of 130 patients were consecutively enrolled. IGF-I measurements were completed for 92 patients due to an unexpected shortage of reagents in the laboratories. One patient was excluded for not meeting the study criteria, leading to 91 patients in the final analysis.

### 2.2 Assessments

A Comprehensive Geriatric Assessment (CGA) was performed for each participant, which included a detailed clinical history, physical examination, electrocardiogram, and a review of all relevant diagnostics. The Cumulative Illness Rating Scale for Geriatrics (CIRS-G) was then completed and reviewed by two physicians. Any discrepancies in the CIRS-G scores were resolved with a third physician. For the analysis, the CIRS-G was calculated excluding the psychiatric item (item 14), resulting in a 13-item scale. Physical activity levels were evaluated using the self-reported Physical Activity Scale for the Elderly (PASE). All patients provided their informed consent, and the study was approved by the local board of Sapienza University of Rome and conformed to the ethical guidelines of the Declaration of Helsinki.

#### 2.2.1 Body composition

After the initial visit, enrolled participants returned to the clinic for anthropometric measurements (i.e., weight, height, and waist circumference). Body composition was assessed using bioimpedance analysis (BIA 101, AKERN, Italy), conducted between 8:30 a.m. and 11:00 a.m. with participants fasted overnight. BIA was performed following a 5-min rest in the supine position, with the participant’s upper limbs abducted at a 30° angle and lower limbs at a 45° angle. Bioelectrical data and body composition parameters were then collected, and reported as follows: Total Body Water (TBW) in liters, Intra- and Extracellular Water (ICW and ECW, respectively) in liters, Fat-Free mass (FFM) in kilograms, Muscle Mass (MM) in kilograms, Body Cell Mass (BCM) in kilograms, and Fat Mass (FM) in kilograms.

#### 2.2.2 Blood tests and biomarkers

Blood samples were collected from the participants and sent to a local laboratory. All samples were handled and analyzed according to standard practices for measuring hematological parameters, including complete blood count, serum electrolytes, metabolic panel, albumin, folate, Vitamin B12, 25(OH) Vitamin D, IGF-I, and C-reactive protein (CRP).

### 2.3 Statistical analysis

Statistical analysis was performed using SPSS software (version 29.0.2; IBM, Armonk, NY, United States). Descriptive statistics were used to summarize the data. Categorical variables were reported as frequencies or percentages, while continuous variables were presented as means and standard deviations (SD) or medians and interquartile ranges (IQR), depending on the distribution of the data. Spearman’s rank correlation was used to assess associations between variables. To compare numerical data between groups, either Student’s t-test or the Mann–Whitney U test was used, based on the normality of the data distribution. All tests were two-tailed, and a *p*-values less than 0.05 was considered statistically significant. To investigate the factors independently associated with FFM, a multivariate linear regression model was constructed using the variables of interest. Prior to analysis, the fundamental assumptions of linear regression were assessed. To test the hypothesis of IGF-I as a mediator in the relationship between Vitamin D and FFM, a mediation analysis was conducted using the model described by Preacher and Hayes (62). Specifically, we employed Model 4 of the SPSS PROCESS Macro to test the indirect effect of the independent variable [25(OH) Vitamin D] on the dependent variable (FFM) through the mediator (IGF-I) (63). The analysis utilized 5,000 bootstrap samples to estimate the indirect effect and construct 95% confidence intervals (CIs) for the mediation effect. Original figures were prepared using GraphPad Prism (version 10.4.1 for Mac, GraphPad Software, Boston, Massachusetts, United States) and BioRender.

## 3 Results

### 3.1 Patient characteristics

The analysis included 91 patients (50.5% women). The mean age of participants was 74.4 ± 7.2 years, and the mean BMI was 28.0 ± 3.9 kg/m^2^. The prevalence of hypertension was 76.9%, while dyslipidemia and Impaired Fasting Glucose (IFG)/T2DM were present in 59.3 and 28% of the participants, respectively. Atrial fibrillation, Coronary Heart Disease (CHD), and history of Transient Ischemic Attacks (TIA) were each present in 6.5% of participants. The mean CIRS-G score was 14.4 ± 4.1 while the severity index was 1.16 ± 0.32, suggesting a moderate level of multimorbidity. Clinical characteristics of patients are reported in Tables 1, 2.

**Table 1:** Patient characteristics and blood parameters.

Parameters	Participants (*N* = 91)
Age, years	74.4 ± 7.2
Females, *n* (%)	46 (50.5)
BMI, kg/m^2^	28 ± 3.9
Body weight, kg	73 ± 12.5
Waist circumference, cm	99.2 ± 10.2
Blood count	
Hemoglobin, g/dL	13.8 ± 1.23
Lymphocytes,10^3^/μL	2 ± 0.83
Platelets, 10^3^/μL	223 (74.5)
Kidney and liver function	
Creatinine, mg/dL	0.88 ± 0.2
eGFR (CKD-EPI), mL/min/1.73 m2	81.67 ± 19.4
AST, U/L	21.3 ± 11.2
ALT, U/L	19.5 ± 16
Hormones, metabolism and inflammation	
Fasting plasma glucose, mg/dL	94.4 ± 17.7
Fasting Insulin, μUI/mL	9.3 (7.5)
Hemoglobin A1c, %	5.6 (0.53)
IGF-I, ng/mL	122 (69.8)
Cholesterol, mg/dL	196.5 ± 33.8
LDL, mg/dL	117.3 ± 33.3
HDL, mg/dL	58 ± 13
Triglycerides, mg/dL	111 ± 40
CRP, μg/L	2000 (2525)
Nutrition and physical activity	
Total Protein, g/dL	71.4 ± 7.9
Albumin, g/dL	4.5 ± 0.2
Folate (ng/mL)	8 (4.1)
Vitamin B12 (pg/mL)	322 (246)
25(OH) Vitamin D (ng/mL)	27.04 ± 14.69
PASE score	116 ± 58.96

Data are presented as mean (± SD) or median (IQR). Glucose, total cholesterol, LDL, HDL, triglycerides, folate, vitamin B12, and Physical Activity Scale for the Elderly (PASE) were available for 88 patients; insulin for 82 patients, AST and ALT were available for 81 patients, C-reactive protein (CRP), 78 patients.

**Table 2:** Prevalence of cardiometabolic disorders and level of multimorbidity.

Clinical characteristics	Participants (*N* = 91)
Hypertension, *n* (%)	70 (76.9)
Dyslipidemia, *n* (%)	54 (59.3)
IFG/T2DM, *n* (%)	26 (28)
TIA, *n* (%)	6 (6.5)
CHD, *n* (%)	6 (6.5)
Atrial Fibrillation, *n* (%)	6 (6.6)
CIRS-G (TS)	14.4 ± 4.1
CIRS-G (SI)	1.16 ± 0.32
Medications*	
Antihypertensives (%)	80
Antiplatelet (%)	62
Oral hypoglycemics (%)	16
Lipid lowering (%)	43

Data are presented as percentages or mean (± SD). CIRS-G (TS): Cumulative Illness Rating scale for geriatrics (Total Score); CIRS-G (SI): Cumulative Illness Rating scale for Geriatrics (Severity Index). IFG, impaired fasting glucose; T2DM, type 2 diabetes mellitus. TIA, transient ischemic attack; CHD, coronary heart disease. *Most prevalent medications prescribed for cardiometabolic disorders were available for 68 patients.

### 3.2 Relationship between IGF-I, vitamin D, and parameters of body composition

**Figure 1:**
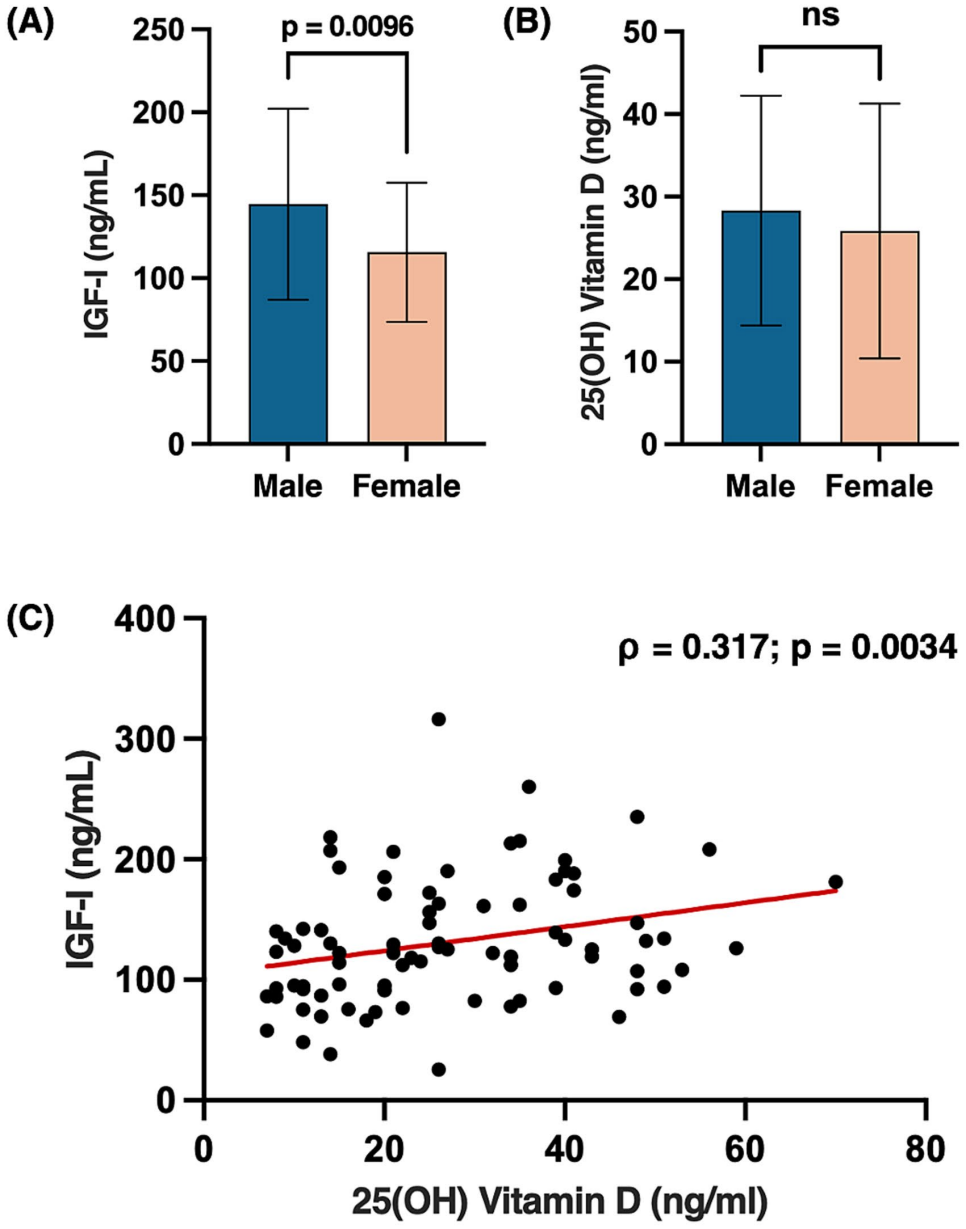
Sex differences in circulating levels of IGF-I **(A)** and 25(OH) Vitamin D **(B)**. Bars represent mean ± SD. Scatter plot showing the Spearman correlation between 25(OH) Vitamin D and IGF-I levels, with a fitted regression line **(C)**; *p*-values < 0.05 were considered statistically significant. Created with BioRender.com. Vicinanza, R. (2025) https://BioRender.com/l25t663.

The median IGF-I level was 122.0 ng/mL (IQR, 69.8), while the mean 25(OH) Vitamin D level was 27.04 ng/mL (±14.69 SD). IGF-I levels were significantly higher in males compared to females (*p* = 0.0096), whereas no significant sex differences were observed for the 25(OH) Vitamin D (Figures 1A,B). Levels of 25(OH) Vitamin D positively correlated with IGF-I (*ρ* = 0.317, *p* = 0.003) (Figure 1C), while no significant correlations were found between 25(OH) Vitamin D and body composition parameters. As expected, parameters of muscularity were higher in males compared to females (ICW: *p* < 0.001; FFM: *p* < 0.001; MM: *p* < 0.001; BCM: *p* < 0.001), while no significant sex differences were observed for FM (*p* = 0.272). In an exploratory sex-stratified analysis, a positive trend was observed between IGF-I and BCM in females only (*ρ* = 0.256, *p* = 0.086). Additionally, a direct correlation between FM and CRP was found in the overall cohort (ρ = 0.387, *p* < 0.001).

Given the lack of consistent sex-specific associations between IGF-I and parameters of muscularity, the cohort was subsequently dichotomized based on the median IGF-I level, resulting in two subgroups: low (IGF-I ≤ 122.0 ng/mL) and high (IGF-I > 122.0 ng/mL). As summarized in Table 3, significant clinical differences between the two groups were observed for number of males (high IGF-I vs. low IGF-I, 29 vs. 18; *p* = 0.017) and 25(OH) Vitamin D levels (high IGF-I vs. low IGF-I, 30.37 ± 15.04 vs. 23.45 ± 2.14 ng/mL; *p* = 0.031).

**Table 3:** Clinical variables according to high (≥122.0 ng/mL) and low (<122.0 ng/mL) levels of IGF-I.

Parameter	High IGF-I ≥122.0 ng/mL	Low IGF-I levels <122.0 ng/mL	*p*-value
	(*N* = 46)	(*N* = 47)	
Age, years	74.0 ± 7.5	74.7 ± 6.9	0.588
Males, *n* (%)	29 (63)	18 (38)	**0.017**
Anthropometrics			
Body mass index, kg/m^2^	27.9 ± 4.1	28.3 ± 4.2	0.669
Body weight, kg	75.2 ± 13.9	72.1 ± 12.3	0.266
Waist circumference, cm	101.3 ± 10.5	98.1 ± 10.6	0.154
Nutrition and hormones			
Folate (ng/mL)	7.70 (4)	7.95 (4.3)	0.941
Vitamin B12 (pg/mL)	361 (277)	337 (249)	0.366
25 OH Vitamin D (ng/mL)	30.37 ± 15.04	23.45 ± 2.14	**0.031**
Albumin, g/dL	45.15 ± 2.34	44.91 ± 2.58	0.658
Fasting plasma glucose, mg/dL	88 (24)	95 (19)	0.382
Fasting Insulin, μUI/mL	9.3 (7.1)	9 (9.53)	0.579
Hemoglobin A1c, %	5.6 (0.70)	5.5 (0.5)	0.342
Physical activity			
PASE score	116.5 ± 63.76	118.6 ± 54.94	0.867
Inflammation and comorbidity			
C-reactive protein, μg/mL	2000 (2700)	2200 (2550)	0.889
CIRS-G (TS)	12.47 ± 0.67	12.37 ± 0.54	0.455
CIRS-G (SI)	1.1 ± 0.3	1.1 ± 0.3	0.956

Data are presented as mean (± SD) or median (IQR). Student’s *t*-test or the Mann–Whitney *U* test was used based on the normality of the data distribution; *p*-values < 0.05 were considered statistically significant and indicated in bold. CIRS-G (TS): Cumulative Illness Rating Scale for Geriatrics (Total Score); CIRS-G (SI): Cumulative Illness Rating Scale for Geriatrics (Severity Index); PASE: Physical Activity Scale for the Elderly.

In terms of body composition, patients with higher IGF-I exhibited significantly greater values, compared to low IGF-I, for the following parameters: TBW (41.69 ± 8.7 Lt vs. 37.70 ± 7.92 Lt; *p* = 0.024), ICW (21.50 [IQR, 8.6] Lt vs. 17.55 [IQR, 9.3] Lt; *p* = 0.018), FFM (52.23 ± 11.06 kg vs. 47.11 ± 9.90 kg; *p* = 0.022), MM (33.26 ± 7.45 kg vs. 29.45 ± 7.52 kg, *p* = 0.017) and BCM (26.66 ± 9.46 kg vs. 24.77 ± 8.90 kg; *p* = 0.046). No significant difference in FM was observed between the two groups (Table 4).

**Table 4:** Body composition parameters according to high and low levels of IGF-I.

Body composition measures	High IGF-I ≥122.0 ng/mL	Low IGF-I levels <122.0 ng/mL	*p*-value
TBW, Lt	41.69 ± 8.7	37.70 ± 7.92	**0.024**
ICW, Lt	21.50 (8.6)	17.55 (9.3)	**0.018**
ECW, Lt	20.06 (7.2)	18.25 (4.28)	0.116
FFM, kg	52.23 ± 11.06	47.11 ± 9.90	**0.022**
MM, kg	33.26 ± 7.45	29.45 ± 7.52	**0.017**
BCM, kg	26.66 ± 9.46	24.77 ± 8.90	**0.046**
FM, kg	25.59 ± 6.83	26.62 ± 7.42	0.491

Data is presented as mean (± SD) or median (IQR). Student’s *t*-test or the Mann–Whitney *U* test was used based on the normality of the data distribution; *p*-values < 0.05 were considered statistically significant and indicated in bold. TBW, total body water; ICW, intracellular water; ECW, extracellular water; FFM, fat-free mass; MM, muscle mass; BCM, body cell mass; FM, fat mass.

Linear regression analysis was performed to identify factors independently associated with FFM. The analysis revealed that IGF-I levels (*B* = 0.40, *p* = 0.034) and male sex (*B* = 13.933, *p* < 0.001) were independently and significantly associated with FFM. No significant associations were found for age (*p* = 0.154), 25(OH) Vitamin D (*p* = 0.398), levels of physical activity (PASE score) (*p* = 0.425), and level of multimorbidity (CIRS-G score) (*p* = 0.645) (Table 5).

**Table 5:** Linear regression analysis with FFM (Fat-Free Mass) as a dependent variable.

Variable	*B*	S. E.	*t*	95% CI		*p*-value
Age, years	−0.193	0.143	−1.441	−0.461	0.074	0.154
Male	13.933	1.872	7.443	10.198	17.667	**<0.001**
IGF-I (ng/mL)	0.40	0.019	2.159	0.003	0.077	**0.034**
25(OH) Vitamin D (ng/mL)	−0.53	0.062	−0.850	−0.177	0.071	0.398
PASE score	−0.13	0.016	−0.802	−0.045	0.019	0.425
CIRS-G (TS)	0.691	1.494	0.463	−2.289	3.671	0.645

CIRS-G (TS), Cumulative Illness Rating Scale for Geriatrics (Total Score); PASE, Physical Activity Scale for the Elderly; *p*-values < 0.05 were considered statistically significant and indicated in bold.

### 3.3 Mediation analysis

**Figure 2:**
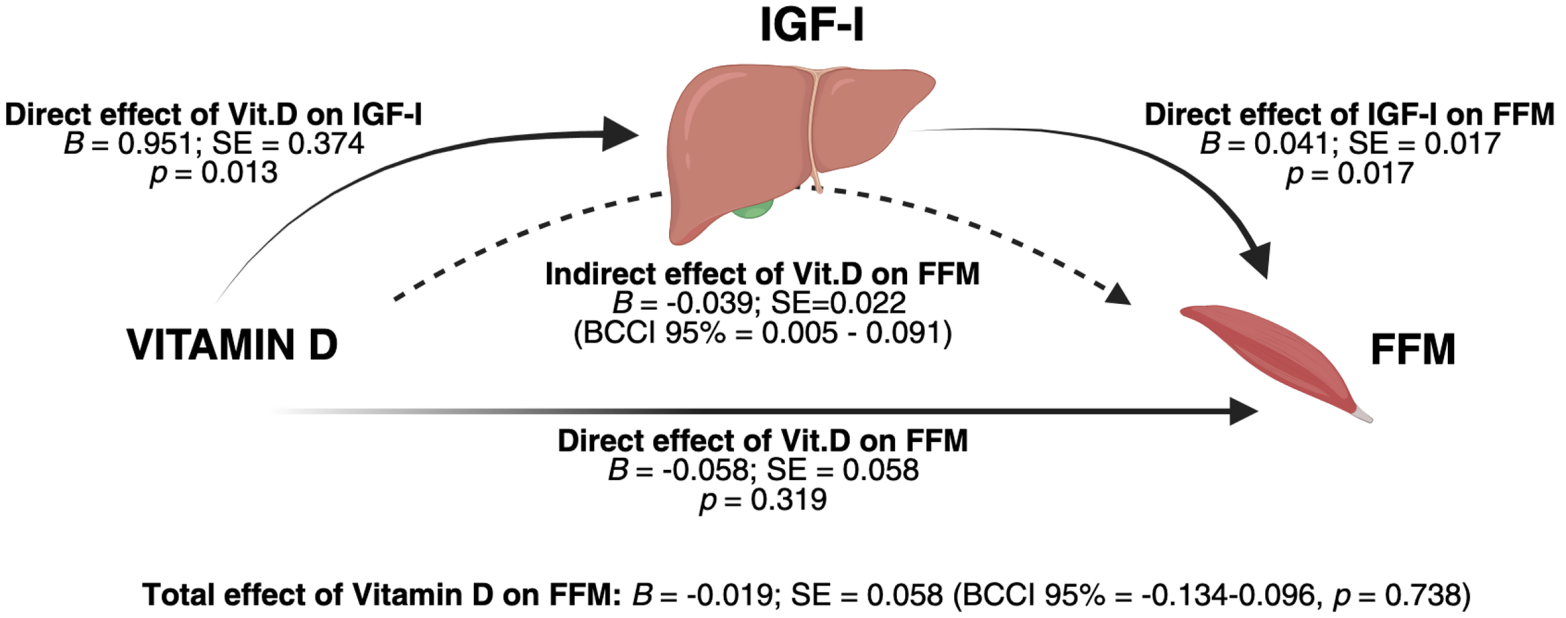
Integrated graphical representation of the mediation model and conceptual framework. Results of the mediation analysis were obtained after controlling for age and sex. The analysis revealed a significant indirect effect of 25(OH) Vitamin D on fat-free mass (FFM) through IGF-I as a mediator, suggesting a physiological regulatory pathway in which Vitamin D influences FFM by modulating IGF-I in the liver. Male sex was a significant covariate in the model (*B* = 12.555, *p* < 0.001). BCCI: Bias-corrected confidence intervals; SE = Standard Error. Created with BioRender.com. Vicinanza, R. (2025) https://BioRender.com/c27j696.

Although our analysis found no significant associations between Vitamin D and FFM, we hypothesized that the effect of Vitamin D on FFM could be mediated by IGF-I. To test this hypothesis, we conducted a mediation analysis, using 25(OH) Vitamin D as the independent variable, FFM as the dependent variable, and IGF-I as the mediator, adjusting for age and sex. The results of the mediation analysis showed a significant positive effect of Vitamin D on IGF-I (*B* = 0.951; SE = 0.374, *p* = 0.013). In turn, IGF-I was positively associated with FFM (*B* = 0.041; SE = 0.017, *p* = 0.017). As expected, the direct effect of Vitamin D on FFM was not significant (*B* = −0.058; SE = 0.058, *p* = 0.319), nor was the total effect of Vitamin D on FFM (*B* = −0.019; SE = 0.058, 95% CI: −0.134, 0.096, *p* = 0.738). However, the indirect effect of Vitamin D on FFM through IGF-I was significant (*B* = 0.039; SE = 0.022, 95% CI: 0.005, 0.091) with male sex being a significant covariate in the model (*B* = 12.555, *p* < 0.001). Results of the mediation analysis are reported in Figure 2, integrated with the associated graphical representation of the theoretical framework.

## 4 Discussion

The present study investigated the relationships between IGF-I, Vitamin D, and body composition parameters in geriatric outpatients with moderate multimorbidity. Our findings showed that (i) IGF-I positively and significantly correlated with Vitamin D, (ii) higher IGF-I, but not Vitamin D, was significantly associated with parameters of muscularity, and along with male sex, was an independent predictor of FFM, (iii) IGF-I was an indirect mediator of the effect of Vitamin D on FFM, although no significant direct or total effects were found.

The observed relationship between Vitamin D and IGF-I was consistent with previous findings (46, 64–66), including a Mendelian randomization study demonstrating a positive association between IGF-I and 25(OH) Vitamin D levels (45). Additionally, a 12-week intervention study with oral Vitamin D3 supplementation resulted in a dose-dependent increase of IGF-I in adults (67), and similar results were found with 6-month supplementation in overweight patients (68). However, a systematic review and meta-analysis of clinical trials yielded mixed results (49). In animal models, VDR knockout mice exhibited reduced IGF-I levels (69), although the precise mechanisms through which Vitamin D may modulate IGF-I production and bioavailability remain unclear (70). *In vitro* studies suggested that non-parenchymal liver cells (e.g., stellate cells, sinusoidal endothelial cells), rather than hepatocytes, express the VDR (52). In turn, the binding of the VDR to the Vitamin D Responsive Element (VDRE) has been shown to activate the promoter regions of the Insulin-like Growth Factor Binding Protein 3 (IGFBP-3) gene, the primary binding protein for IGF-I, in several cell types (71, 72). On the other hand, IGF-I has been shown to stimulate 1α-hydroxylase enzyme in renal cells, while treatment with IGF-I has been reported to significantly increase Vitamin D levels in healthy individuals (55). Taken together, these studies support the existence of a mutual nutrient-hormone interaction that should be considered in clinical practice, particularly in the assessment of Vitamin D deficiency, opening new windows of opportunity to explore treatment strategies.

Regarding the second aim of the study, consistent with previous observations (10, 37), we found that higher IGF-I levels were associated with TBW, ICW, MM, BCM and FFM. Supporting the role of IGF-I in maintaining healthy body composition, particularly in older adults, the InChianti study demonstrated that lower IGF-I concentrations were associated with an increased risk of sarcopenia (73). Other studies have shown that reduced serum concentrations of IGF-I correlated with increased frailty measures (74), decreased functional outcomes (75–78), and a reduced number of motor units (79). However, the exploratory analysis of variables found no significant association between IGF-I and the PASE scores, likely due to the low levels of physical activity within the cohort (80, 81). As expected, CRP was associated with FM (82, 83).

The relationship between IGF-I and clinical outcomes is complex, as both low and high IGF-I concentrations have been associated with increased risk of cancer, CVD, and all-cause mortality (39, 84). Rahmani et al. suggested that IGF-I levels between 120 and 160 ng/mL were associated with positive clinical outcomes and reduced mortality risk (85). Notably, consistent with this observation, in our cohort the median IGF-I value was 122 ng/mL, and no significant associations were found between IGF-I and levels of multimorbidity evaluated with the CIRS-G. Moreover, only participants with no history of cancer in the past 5 years were included, allowing this research to focus primarily on the role of IGF-I on body composition.

Although no associations were found between Vitamin D and parameters of muscularity in our cohort, such evidence has been observed in both human studies and animal models (24, 58, 86, 87). Therefore, we hypothesized a nutrient-hormone interaction where Vitamin D stimulates IGF-I in the liver, which in turn could mediate the effect of Vitamin D on FFM. Using a mediation analysis, previously implemented in the context of multimorbidity (88), we found that IGF-I was an indirect-only mediator for the effect of Vitamin D on FFM, while no significant direct or total effects were observed. Although speculative, these findings suggest a potential regulatory pathway through which Vitamin D may influence lean body mass via IGF-I. Thus, considering this pathway in the clinical setting could further support the evaluation of nutritional status and the assessment of pre-frailty.

Regarding the methods used for this statistical model, we acknowledge that in a typical mediation analysis, a significant relationship between the independent and dependent variable is generally considered a prerequisite for examining the indirect effect (89). Nevertheless, according to Rucker et al. and Preacher and Hayes, the absence of a significant direct or total effect does not preclude the investigation into the indirect effect if the theoretical framework or the rationale behind the analysis supports its plausibility (90, 91). Therefore, considering the described roles of both IGF-I and Vitamin D on lean body mass, we developed an integrated model to illustrate the mediation analysis results alongside the proposed physiological regulatory pathway that may underlie the observed associations (Figure 2).

However, this study has several limitations. First, the cross-sectional design did not allow for the determination of causality or the directionality of the observed associations. Second, the relatively small sample size constrained our ability to perform statistically robust stratifications by age and sex, limiting further investigation of subgroup differences without the loss of statistical power. Consequently, we conducted exploratory sex-stratified analyses with a limited number of participants and low intra-group variance, which may reduce the generalizability and reproducibility of our findings compared to larger studies. Furthermore, although free IGF-I is widely used to measure its serum levels (92), a more comprehensive evaluation of IGF-I bioavailability should also include IGFBP-3. Another limitation is the reliance on a self-administered questionnaire to evaluate physical activity levels, although we inferred the physical function of participants from indices of muscularity, measured with the BIA, and combined with assessment of ADLs and IADLs. Additionally, dietary intake was only partially collected with medical history, and more detailed information about the intake of macronutrients should be addressed. On the other hand, when the participants were divided by IGF-I levels, no significant clinical differences were found between the two groups aside from the variables of interest, indicating a relatively homogeneous sample. Moreover, examining the role of IGF-I in patients with multimorbidity offered a more real-life approach, particularly in geriatric medicine, since research in this population is often underrepresented.

In conclusion, these findings provide additional evidence of (i) a positive correlation between IGF-I and Vitamin D and (ii) the positive effect of IGF-I on parameters of muscularity. Finally, the mediation model suggests that IGF-I may contribute to the physiological effect of Vitamin D on FFM, with possible implications for assessing pre-frailty and personalizing nutrition interventions in this demographic.

Further research is needed to explore the underlying mechanisms and establish causality of these relationships.

## Data Availability

The raw data supporting the conclusions of this article will be made available by the authors, without undue reservation.
